# 

**Published:** 1875-12

**Authors:** 


### CONTENTS:

### ORIGINAL COMMUNICATIONS.

ARTICLE I.—Proceedings of the American Academy of Dental Surgery.......... 337

ARTICLE II.—Pyæmia the Result of Alveolar Abscess. By W. T. Crutchfield, M. D. 346

### SELECTED ARTICLES.

ARTICLE III.—Teeth and Toothache.......... 347

ARTICLE IV.—Suggestions. By W. C. Eastlake, D. D. S.......... 354

ARTICLE V.—Proceedings of the American Dental Association. (Continued.)..... 361

### EDITORIAL, ETC.

A Reply.......... 379

Death while Unconscious from Ether.......... 379

Correction.......... 379

Virginia State Dental Asssociation.......... 379

### MONTHLY SUMMARY.

On the Diseases Ascribed to Dentition and their Pathological Admissibility........ 382
Third Dentition.......... 382
Syphilis of Parents Affecting the Offspring.......... 383
Curious Brain Lesions.......... 383
Neuralgia Treated by Nerve-Stretching.......... 384
Relief of a Toothache by Bicarbonate of Soda.......... 384
Dental Neuralgia.......... 384

